# Distinct Patterns of Rhizosphere Microbiota Associated With Rice Genotypes Differing in Aluminum Tolerance in an Acid Sulfate Soil

**DOI:** 10.3389/fmicb.2022.933722

**Published:** 2022-06-17

**Authors:** Xun Xiao, Jia Lin Wang, Jiao Jiao Li, Xiao Li Li, Xin Jun Dai, Ren Fang Shen, Xue Qiang Zhao

**Affiliations:** ^1^State Key Laboratory of Soil and Sustainable Agriculture, Institute of Soil Science, Chinese Academy of Sciences, Nanjing, China; ^2^University of Chinese Academy of Sciences, Beijing, China; ^3^College of Land Resource and Environment, Jiangxi Agricultural University, Nanchang, China

**Keywords:** aluminum toxicity, acid sulfate soil, rice genotypes, microbial community structure, co-occurrence network

## Abstract

Rhizosphere microbes are important for plant tolerance to various soil stresses. Rice is the most aluminum (Al)-tolerant small grain cereal crop species, but the link between rice Al tolerance and rhizosphere microbiota remains unclear. This study aimed to investigate the microbial community structure of aluminum-sensitive and Al-tolerant rice varieties in acid sulfate soil under liming and non-liming conditions. We analyzed the rice biomass and mineral element contents of rice plants as well as the chemical properties and microbial (archaea, bacteria, and fungi) communities of rhizosphere and bulk soil samples. The results showed that the Al-tolerant rice genotype grew better and was able to take up more phosphorus from the acid sulfate soil than the Al-sensitive genotype. Liming was the main factor altering the microbial diversity and community structure, followed by rhizosphere effects. In the absence of liming effects, the rice genotypes shifted the community structure of bacteria and fungi, which accounted for the observed variation in the rice biomass. The Al-tolerant rice genotype recruited specific bacterial and fungal taxa (*Bacillus*, *Pseudomonas*, *Aspergillus*, and *Rhizopus*) associated with phosphorus solubilization and plant growth promotion. The soil microbial co-occurrence network of the Al-tolerant rice genotype was more complex than that of the Al-sensitive rice genotype. In conclusion, the bacterial and fungal community in the rhizosphere has genotype-dependent effects on rice Al tolerance. Aluminum-tolerant rice genotypes recruit specific microbial taxa, especially phosphorus-solubilizing microorganisms, and are associated with complex microbial co-occurrence networks, which may enhance rice growth in acid sulfate soil.

## Introduction

Aluminum (Al) is the most abundant metal element in the earth’s crust. Most of the Al in soil exists as insoluble aluminosilicates and aluminum oxides, which do not adversely affect plant growth. However, if the soil pH decreases to less than 5.0, Al ions are released from clay minerals and inhibit root growth, which restricts the ability of plants to obtain water and nutrients (Ma et al., 2001; Kunito et al., 2016). Thus, Al toxicity is the major factor limiting plant productivity in acidic soils, which account for approximately 30% of the ice-free land area worldwide (Von Uexküll and Mutert, 1995; Zhao et al., 2014).

To mitigate Al toxicity, plants have evolved multiple mechanisms mediating Al exclusion and Al tolerance (Kochian et al., 2005; Ma, 2007), among which the secretion of organic acids (e.g., malic acid, oxalic acid, and citric acid) from plant roots is considered to be the main mechanism (Ma et al., 2001). Some root exudates can form non-toxic compounds with Al ions in the rhizosphere (Kochian et al., 2005), leading to decreased Al bioavailability and toxicity to various soil organisms. These reactions result in variations between the bulk soil and the rhizosphere of Al-tolerant and Al-sensitive plants. Plant species can affect the rhizosphere microbial community (Costa et al., 2006; Bulgarelli et al., 2013). Therefore, the rhizosphere microbial community of Al-tolerant plants may differ from that of Al-sensitive plants.

Plant-specific root exudation modulates the microbial community in the rhizosphere, which affects microbial diversity and activity (Bulgarelli et al., 2013; Compant et al., 2019). The rhizosphere microbiome may enhance various plant characteristics, including nutrient acquisition, stress tolerance, and defense against pathogens (Hu et al., 2018; Mendes et al., 2018; Gu et al., 2020; Benitez et al., 2021). When subjected to environmental stresses, plant roots may recruit specific microbes to alleviate these stresses (Rodriguez et al., 2019). For example, some plant-beneficial bacteria and fungi (e.g., *Pseudomonas*, *Rhizobium*, *Fusarium*, *Penicillium*, and *Aspergillus*) recruited by plant roots can produce organic acids or trigger the production of root exudates (Phillips et al., 2004; Kiers et al., 2011), thereby protecting plants from the toxic effects of Al (Yang et al., 2012a). Some Al-tolerant bacteria, such as *Enterobacter*, *Klebsiella*, *Serratia*, and *Stenotrophomonas*, can alleviate Al toxicity via the formation of Al^3+^ siderophore complexes and the improvement of plant phosphorus (P) uptake (de la Luz Mora et al., 2017). Therefore, clarifying the relationship between the rhizosphere microbial community and plant Al tolerance is critical for increasing plant productivity and sustainable agriculture in acidic soils.

Acid sulfate soil contains considerable amounts of reduced sulfur compounds (mainly pyrite) that produce sulfuric acid upon their oxidation and subsequently increase soil acidification and Al phytotoxicity. Acid sulfate soil-derived environmental degradation is a common phenomenon worldwide in coastal lowland areas, particularly in tropical and subtropical regions (Lin, 1999). Climate conditions (e.g., rainfall and temperature) in regions with acid sulfate soil are favorable for rice (*Oryza sativa* L.) cultivation, but rice production has been limited by the poor conditions of acid sulfate soil (Lin, 1999; Zhang et al., 2020). Rice, which is a staple food for half of the global population, is the most Al-tolerant species among small grain cereal crops, but this Al tolerance varies among rice genotypes (Famoso et al., 2010; Zhao et al., 2013). Plant genotypes can affect rhizosphere physicochemical properties and alter rhizosphere microbiomes (Peiffer et al., 2013; Walters et al., 2018). Soil Al stress leads to distinct rhizosphere bacterial and fungal community compositions that differ between Al-tolerant and Al-sensitive genotypes of dryland crops, including wheat (*Triticum aestivum* L.) and soybean (*Glycine max* L.) (Yang et al., 2012a,b; Wang et al., 2013; Lian et al., 2019; Shi et al., 2020a,b). These reports suggest that Al-tolerant genotypes may recruit certain microbial taxa to mitigate Al toxicity. However, there is a lack of information on how the rhizosphere microbial community is affected by rice genotypes differing in Al tolerance in flooded paddy soil. Aluminum-tolerant rice and dryland plants may differ in terms of the microbes they recruit in the rhizosphere under Al stress conditions because of the diversity in hydrological conditions. Moreover, previous studies on dryland plants focused on individual bacteria or fungi. However, archaea, bacteria, and fungi all play important roles in plant nutrient acquisition and stress tolerance (Yang et al., 2009; Zhang et al., 2019; Ayangbenro and Babalola, 2021; Naitam and Kaushik, 2021). Thus, a comprehensive examination of bacterial, fungal, and archaeal communities in the rhizospheres of Al-sensitive and Al-tolerant rice genotypes is required to clarify how rice Al tolerance is influenced by rhizosphere microbes in acid sulfate soil.

In this study, we grew two rice varieties differing in Al tolerance in acid sulfate soil with or without liming and examined the mineral element contents of rice plants and the microbial (archaea, bacteria, and fungi) communities in bulk and rhizosphere soils. Lime was used to increase the soil pH and decrease the Al-exchangeable and toxic fraction in the soil. We hypothesized that changes in the rhizosphere microbial community related to rice Al tolerance are associated with rice nutrient uptake and growth in acid sulfate soils.

## Materials and Methods

### Soil and Plant Materials

The acid sulfate soil used in this study was collected from acidic farmland (20 cm depth) located in Chonglou town, Taishan city, Guangdong province, China (22°06′55′′N, 112°46′32′′E). The collected soil samples were air-dried and filtered through a 2-mm sieve after removing roots and stones. The soil properties were as follows: pH, 3.97; alkali-hydrolyzable nitrogen (N), 159.8 mg kg^–1^; available P, 2.80 mg kg^–1^; available potassium (K), 93.5 mg kg^–1^; organic carbon (C), 23.6 g kg^–1^; exchangeable Al, 230.06 mg kg^–1^; organic bound Al, 184.80 mg/kg; clay content, 39.17%; silt content, 50.67%; and sand content, 10.16%. Soil mineral compositions were as follows: vermiculite, 1.33%; hydromica, 4.67%; kaolinite, 14.67%; chlorite, 6.33%; gibbsite, 1.00%; quartz, 68.67%; and feldspar, 3.33%. The two rice varieties used in this study were Koshihikari (Al-tolerant genotype) and Kasalath (Al-sensitive genotype) (Ma et al., 2002; Zhao et al., 2013).

### Experimental Design and Sampling

A pot experiment was conducted at the Institute of Soil Science, Chinese Academy of Sciences, Nanjing, China (32°03′40′′N, 118°47′58′′E), to examine the following two factors: (1) rice genotype (Al-tolerant or Al-sensitive) and (2) soil management (limed or non-limed). The experiment was performed using a randomized complete block design with four treatments: Al-S (Al-sensitive genotype), Al-T (Al-tolerant genotype), Al-S + Ca (Al-sensitive genotype and CaCO_3_), and Al-T + Ca (Al-tolerant genotype and CaCO_3_). Each treatment included six replicates (i.e., 24 pots in total). All pots received the same fertilizer comprising N (urea, 100 mg N kg^–1^), P (KH_2_PO_4_, 50 mg P kg^–1^), and K (KCl, 100 mg K kg^–1^). Half of the pots were amended with lime (CaCO_3_, 3 g kg^–1^). To ensure the homogeneous distribution of fertilizer in each pot, the fertilizer and soil in individual pots were weighed and mixed thoroughly. Each pot (15-cm height, 15-cm top diameter, and 12-cm bottom diameter) contained 2.5 kg of air-dried soil.

Rice seeds were surface-sterilized in 10% H_2_O_2_ solution for 30 min and then thoroughly washed with deionized water. The sterilized seeds were maintained in deionized water for 24 h and then transferred to a net floating on a 500 μM CaCl_2_ solution. After the rice seeds germinated, the seedlings were cultured in a 3.5-L plastic pot containing half-strength Kimura B solution. At the three-leaf stage, rice seedlings were transplanted into potting soil, with three plants per pot. All pots were incubated under a normal diel light cycle in a growth chamber [12-h day (30 ± 1°C):12-h night (23 ± 1°C); 65 ± 5% humidity; 900 μmol m^–2^ s^–1^ illumination intensity]. Rice plants were irrigated daily during the experiment. After 30 days, rice plants were harvested at the tillering stage. The harvested plants were separated into shoots and roots, dried at 75°C to a constant weight, and then ground to a powder for an analysis of the mineral elements. Rhizosphere and bulk soil samples were collected immediately after rice plants were harvested. After shaking the roots for 30 s, we collected the soil adhered to the roots that was defined as rhizosphere soil here. Three rhizosphere soil subsamples were collected for each pot and thoroughly mixed to produce a single sample. Similarly, three bulk soil (i.e., root-free soil) subsamples from individual pots were combined. All soil samples were divided into two parts. One part was frozen and stored at −80°C for DNA extraction, whereas the other part was stored at 4°C before analyzing the soil chemical properties.

### Analyses of Plant and Soil Samples

Ground plant samples were digested in H_2_SO_4_–H_2_O_2_, after which the N concentration in the digestion solution was determined according to the Kjeldahl method (Lu, 1999). Ground plant samples were also digested in HClO_4_–HNO_3_ (1:4). After diluting the digestion solution appropriately, the concentrations of other mineral elements (P, K, Na, Ca, Mg, Fe, Mn, Al, and S) were determined using the Optima 8000 inductively coupled plasma–atomic emission spectrophotometry (ICP-AES) system (PerkinElmer, Waltham, MA, United States).

Soil texture (sand, clay, and silt contents) was determined by Laser Particle Sizer (LS13320, Beckman Coulter Inc., California, United States). Soil mineral composition and contents were measured by X-ray diffractometer (Ultima IV, Rigaku Corporation, Tokyo, Japan). Soil chemical properties were measured according to the methods described by Lu (1999). Briefly, soil pH was measured using the PB-21 pH meter (Sartorius, Göttingen, Germany) and a soil:water suspension (1:2). Soil available P (AP) was extracted using HCl–NH_4_F and analyzed according to the molybdenum blue method. Soil available K was extracted using 1 M CH_3_COONH_4_ and analyzed using the FP640 flame photometer (Shanghai Precision and Scientific Instrument, Shanghai, China). Soil organic C was analyzed according to the dichromate oxidation method. Soil alkali-hydrolyzable N was analyzed using the alkaline hydrolysis diffusion method. Soil exchangeable Al was extracted using 1 M KCl (soil:water, 1:25), whereas exchangeable Ca, Mn, and Mg were extracted using 1 M CH_3_COONH_4_ (soil:water, 1:10). Soil organic bound Al was extracted with 0.1 M CuCl_2_ + 0.5 M KC1 (soil:water, 1:10) as described by Soon (1993). The Al, Ca, Mg, and Mn contents in the extraction solution were determined by ICP-AES (Optima 8000). Soil NH_4_^+^–N and NO_3_^–^–N were extracted using 2 M KCl and then measured by indophenol blue colorimetry and dual-wavelength spectrophotometry methods, respectively.

### Soil DNA Extraction

Soil DNA was extracted using the Fast DNA SPIN Kit for Soil (MP Biomedicals, Santa Ana, CA). The quality and concentration of the extracted DNA were checked using the ND-1000 spectrophotometer (NanoDrop Technologies, Wilmington, United States).

### Illumina MiSeq Sequencing and Quantitative PCR

The diversity of archaeal, bacterial, and fungal communities was investigated on the basis of high-throughput sequencing analyses of the 16S rRNA gene within the archaeal and bacterial V4–V5 region and the ITS rRNA operon within the fungal ITS region. The primers used for amplifying archaeal, bacterial, and fungal sequences and thermal cycling conditions are listed in Supplementary Table 1. The PCR amplification was completed in a 25-μL solution comprising 5 μL 5 × FastPfu Buffer, 5 μL 5 × GC buffer, 2 μL 2.5 mM dNTPs, 1 μL each primer (10 μM), 2 μL template DNA, 8.75 μL ddH_2_O, and 0.25 μL Q5 DNA polymerase. Amplicons were separated and visualized by gel electrophoresis, purified using the AxyPrep DNA Gel Extraction kit (Axygen Biosciences, Union City, CA, United States), and quantified using the QuantiFluor-ST system (Promega, United States). Equal amounts of the purified and quantified amplicons were pooled and then sequenced by Shanghai Personal Biotechnology Co., Ltd. (Shanghai, China) using the Illumina MiSeq PE300 platform (Illumina, San Diego, CA, United States).

The quantitative PCR (qPCR) analysis was performed using the same primers as those used for the high-throughput sequencing to determine the copy numbers of archaeal and bacterial 16S rRNA genes and the fungal ITS gene. Briefly, 20-μL qPCR reaction mixtures, which included 10 μL 2 × SYBR real-time PCR premixture, 0.4 μL each primer, 1 μL soil DNA, and 8.2 μL sterilized water, were prepared. The qPCR analysis was completed using the LightCycler 480 Real-time PCR system (Roche Diagnostics, Mannheim, Germany). The qPCR program was as follows: 95°C for 5 min; 40 cycles of 95°C for 15 s and 60°C for 30 s; melting curve analysis. A standard curve for each target gene was generated using plasmid DNA. The PCR efficiency ranged from 90.94 to 96.45% (*R*^2^ > 0.995).

### Processing of Sequencing Data

Raw FASTQ files were processed using QIIME (version 1.9.1) (Caporaso et al., 2010), QIIME2 (Bolyen et al., 2019), USEARCH (Edgar, 2010), and a GitHub online script (Liu et al., 2021). Briefly, the Illumina sequenced paired-end reads were evaluated using FastQC 0.11.5 (Andrews, 2010). The forward and reverse reads were merged using USEARCH, and then, the low-quality reads (error rate > 1% and redundant) were removed after the barcode and primers were trimmed. All filtered sequences were clustered into amplicon sequence variants (ASVs) on the basis of 100% similarity using unoise3. The taxonomic classifications of the 16S rRNA and ITS gene sequences were determined by screening the Silva132 16S rRNA^1^ and Unite8.0 ITS^2^ databases, respectively, using the RDP classifier algorithm^3^. Finally, 2,378,538, 2,107,800, and 1,812,288 high-quality sequences were obtained for archaea, bacteria, and fungi, respectively. The raw data have been submitted to the NCBI Sequence Read Archive under the following accession numbers PRJNA772948 (archaea), PRJNA773189 (bacteria), and PRJNA773227 (fungi).

### Statistical Analysis

The SPASS 22.0 program was used to perform a two-way ANOVA followed by Duncan’s multiple range test (*P* < 0.05) to detect significant differences in the rice biomass, soil properties, microbial α-diversity, and plant mineral element contents among treatments. A hierarchical cluster analysis, as well as a non-metric multidimensional scaling (NMDS) analysis, based on the Bray–Curtis distance and a PERMANOVA test, were conducted using the “vegan” package in R (version 4.0.5) (Oksanen et al., 2013) to reveal significant differences in the β-diversity of archaeal, bacterial, and fungal community compositions among treatments. A Mantel test was performed using the “vegan” package in R to examine the association between the microbial community and soil properties. The “RandomForest” package in R was used to evaluate the contribution of soil properties, plant mineral elements, and microbial properties to the plant biomass. To analyze genotype-specific ASVs, the R package “edger” was used to identify the differentially abundant ASVs between rice genotypes (Robinson et al., 2010).

### Microbial Co-occurrence Networks

We constructed four networks to analyze the co-occurrence of archaeal, bacterial, and fungal communities for two rice varieties under liming and non-liming conditions. Pairwise correlations between ASVs were obtained by calculating Pearson’s correlation coefficients using the “WGCNA” package in R. We filtered the ASVs present in fewer than two samples. In addition, links with Pearson’s correlation coefficients | *r*| < 0.9 and *P* > 0.001 were eliminated. The nodes within the top 1% of each microbial network were designated as keystone ASVs (Hartman et al., 2018). The networks were visualized using Gephi (version 0.9.2) (Bastian et al., 2009), whereas topological properties were determined using the “igraph” package in R to elucidate community structure differences among networks.

## Results

### Rice Biomass, Mineral Element Contents, and Soil Properties

**FIGURE 1:**
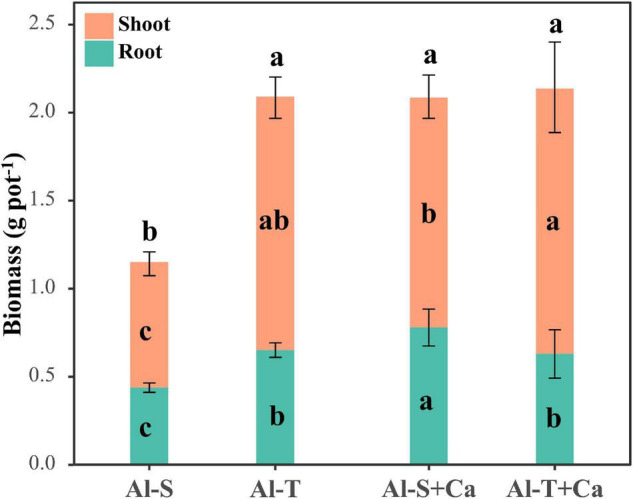
Rice shoot and root biomasses. Different lowercase letters within the bottom, middle, and top parts of columns indicate significant differences in root, shoot, and total biomasses among treatments, respectively (*P* < 0.05). Al-S, Al-sensitive rice; Al-T, Al-tolerant rice; Al-S + Ca, Al-sensitive rice and CaCO_3_; Al-T + Ca, Al-tolerant rice and CaCO_3_.

The total biomass was similar between Al-sensitive and Al-tolerant rice genotypes under the liming condition (Figure 1). Compared with the liming condition, the shoot and root biomasses decreased significantly in the Al-sensitive rice genotype under the non-liming condition (*P* < 0.05) (Figure 1), implying that the Al-tolerant genotype was more tolerant to acid sulfate soil than the Al-sensitive genotype. The P contents of the shoots and roots were higher for the Al-tolerant genotype than for the Al-sensitive genotype under both liming and non-liming conditions (Table 1). Liming significantly increased the shoot and root P contents of only the Al-sensitive genotype (*P* < 0.05). Moreover, the shoot S content was lower for the Al-tolerant genotype than for the Al-sensitive genotype under both liming and non-liming conditions. Liming significantly decreased the shoot S content of the Al-sensitive genotype but had the opposite effect on the shoot S content of the Al-tolerant genotype (*P* < 0.05). The Al-sensitive genotype accumulated more Al, Fe, Mg, and Ca in the roots than the Al-tolerant genotype under both liming and non-liming conditions.

**TABLE 1:** Mineral element contents of rice plants (g⋅kg^–1^).

Elements	Shoots				Roots			
	Al-S	Al-T	Al-S + Ca	Al-T + Ca	Al-S	Al-T	Al-S + Ca	Al-T + Ca
N	3.11 ± 0.88ab	2.45 ± 0.76b	3.03 ± 0.74ab	3.69 ± 0.80a	3.78 ± 0.25a	4.25 ± 0.76a	4.47 ± 1.02a	4.69 ± 0.74a
P	0.77 ± 0.08c	1.42 ± 0.06a	1.00 ± 0.13b	1.55 ± 0.18a	0.44 ± 0.04c	0.78 ± 0.08a	0.66 ± 0.08b	0.81 ± 0.05a
K	28.06 ± 1.91a	27.4 ± 3.28a	31.32 ± 1.39a	28.73 ± 5.86a	13.51 ± 3.06a	14.73 ± 2.37a	14.54 ± 1.16a	15.33 ± 1.6a
Ca	1.31 ± 0.15b	1.69 ± 0.11b	3.26 ± 0.61a	3.69 ± 0.55a	0.98 ± 0.15b	0.55 ± 0.03c	1.37 ± 0.15a	0.97 ± 0.11b
Mg	1.36 ± 0.10b	1.46 ± 0.09b	1.47 ± 0.14b	1.68 ± 0.18a	0.84 ± 0.11b	0.63 ± 0.06c	0.96 ± 0.06a	0.83 ± 0.06b
Na	1.93 ± 0.38a	1.73 ± 0.2a	1.87 ± 0.35a	1.55 ± 0.22a	2.92 ± 0.65b	3.72 ± 0.85a	3.06 ± 0.4ab	3.72 ± 0.38a
S	8.64 ± 0.68a	5.45 ± 0.14d	7.50 ± 0.51b	6.41 ± 0.74c	38.74 ± 4.11a	34.72 ± 3.80a	38.18 ± 2.83a	39.53 ± 5.69a
Fe	0.52 ± 0.12a	0.57 ± 0.15a	0.5 ± 0.16a	0.56 ± 0.09a	0.1 ± 0.04a	0.02 ± 0.00c	0.07 ± 0.03b	0.02 ± 0.00c
Mn	0.17 ± 0.03c	0.23 ± 0.03b	0.2 ± 0.03bc	0.34 ± 0.06a	3.59 ± 0.15b	3.94 ± 0.08a	3.92 ± 0.11a	3.87 ± 0.09a
Al	0.62 ± 0.19b	0.82 ± 0.14a	0.57 ± 0.13b	0.83 ± 0.12a	2.37 ± 0.11a	1.63 ± 0.32c	1.98 ± 0.21b	1.51 ± 0.39c

*Different lowercase letters indicate significant differences among treatments (P < 0.05, Duncan’s multiple range test).*

*Al-S, Al-sensitive rice; Al-S + Ca, Al-sensitive rice and CaCO_3_; Al-T, Al-tolerant rice; Al-T + Ca, Al-tolerant rice and CaCO_3_.*

Liming significantly increased the soil pH and exchangeable Ca content, but decreased the soil exchangeable Al content in the bulk and rhizosphere soils of both genotypes (*P* < 0.05) (Table 2). Interestingly, under the non-liming condition, the AP of the bulk soil was lower for the Al-sensitive genotype than for the Al-tolerant genotype, but the AP of the rhizosphere soil was higher for the Al-sensitive genotype than for the Al-tolerant genotype. This suggested that the Al-tolerant genotype took up more P from the rhizosphere soil than the Al-sensitive genotype.

**TABLE 2:** Soil chemical properties.

Soil properties	Bulk soil				Rhizosphere soil			
	Al-S	Al-T	Al-S + Ca	Al-T + Ca	Al-S	Al-T	Al-S + Ca	Al-T + Ca
pH	4.45 ± 0.03b	4.42 ± 0.02b	5.11 ± 0.05a	5.08 ± 0.04a	4.21 ± 0.07b	4.22 ± 0.06b	4.97 ± 0.10a	4.89 ± 0.10a
Al (mg kg^–1^)	165.16 ± 20.54b	272.89 ± 19.12a	40.87 ± 9.55d	63.78 ± 17.49c	386.69 ± 30.55a	408.94 ± 24.05a	99.84 ± 38.53b	96.55 ± 29.09b
Ca (mg kg^–1^)	60.26 ± 8.39c	130.58 ± 5.13b	656.76 ± 149.75a	733.33 ± 25.69a	139.62 ± 12.79c	126.90 ± 7.99c	748.39 ± 57.00a	692.11 ± 55.19b
Mg (mg kg^–1^)	17.48 ± 2.59c	30.75 ± 2.49a	20.12 ± 1.43b	31.37 ± 1.36a	32.11 ± 4.04a	30.16 ± 1.31a	31.81 ± 1.85a	30.03 ± 1.08a
Mn (mg kg^–1^)	2.48 ± 0.27a	2.43 ± 0.38a	2.05 ± 0.13b	1.87 ± 0.17b	1.66 ± 0.25ab	1.93 ± 0.25a	1.71 ± 0.29ab	1.55 ± 0.33b
AP (mg kg^–1^)	4.41 ± 0.6b	5.62 ± 0.47a	3.98 ± 1.16b	4.34 ± 0.79b	5.17 ± 2.50a	3.07 ± 0.41b	2.96 ± 0.55b	2.15 ± 0.28b
NH_4_^+^-N (mg kg^–1^)	15.62 ± 0.64a	15.88 ± 1.89a	15.46 ± 1.26a	15.07 ± 1.12a	12.86 ± 1.52ab	11.24 ± 1.79b	14.31 ± 1.24a	13.24 ± 0.92a
NO_3_^–^-N (mg kg^–1^)	0.34 ± 0.43a	0.29 ± 0.54a	0.66 ± 0.52a	0.73 ± 0.87a	0.67 ± 0.56a	0.34 ± 0.95a	1.00 ± 0.65a	0.74 ± 0.31a

*Different lowercase letters indicate significant differences among treatments (P < 0.05, Duncan’s multiple range test).*

*Al-S, Al-sensitive rice; Al-S + Ca, Al-sensitive rice and CaCO_3_; Al-T, Al-tolerant rice; Al-T + Ca, Al-tolerant rice and CaCO_3_; AP, available P. Al, Ca, Mg, and Mn referred to soil exchangeable Al, Ca, Mg, and Mn, respectively.*

### Microbial Diversity, Abundance, and Community Structure

**FIGURE 2:**
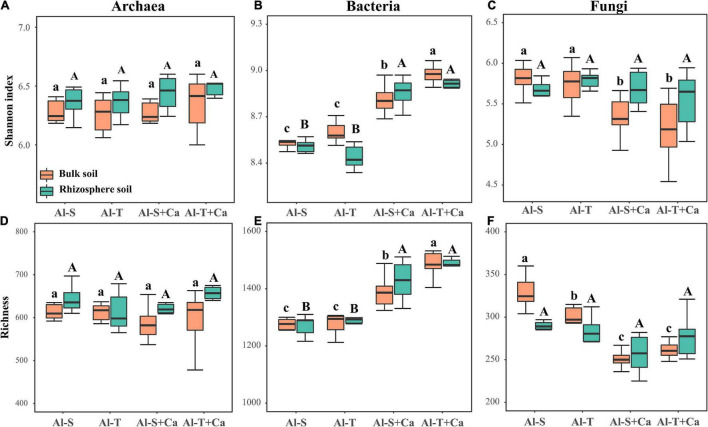
α-diversity of archaea **(A,D)**, bacteria **(B,E)**, and fungi **(C,F)** according to the Shannon and richness indices. Different lowercase and uppercase letters above boxplots indicate significant differences among treatments for the bulk soil and rhizosphere soil (*P* < 0.05), respectively. Al-S, Al-sensitive rice; Al-T, Al-tolerant rice; Al-S + Ca, Al-sensitive rice and CaCO_3_; Al-T + Ca, Al-tolerant rice and CaCO_3_.

Liming did not significantly affect the α-diversity of archaea across all soil samples (Figures 2A,D). Liming increased the Shannon index and richness of bacteria in both bulk and rhizosphere soils (Figures 2B,E), whereas it decreased the Shannon index and richness of fungi, but only in the bulk soil (Figures 2C,F). However, there was no significant difference in the α-diversity of archaea, bacteria, and fungi between the Al-tolerant and Al-sensitive genotypes. The abundance of archaea, bacteria, and fungi was not significantly affected by liming, soil compartments, or rice genotypes (Supplementary Figure 1).

**FIGURE 3:**
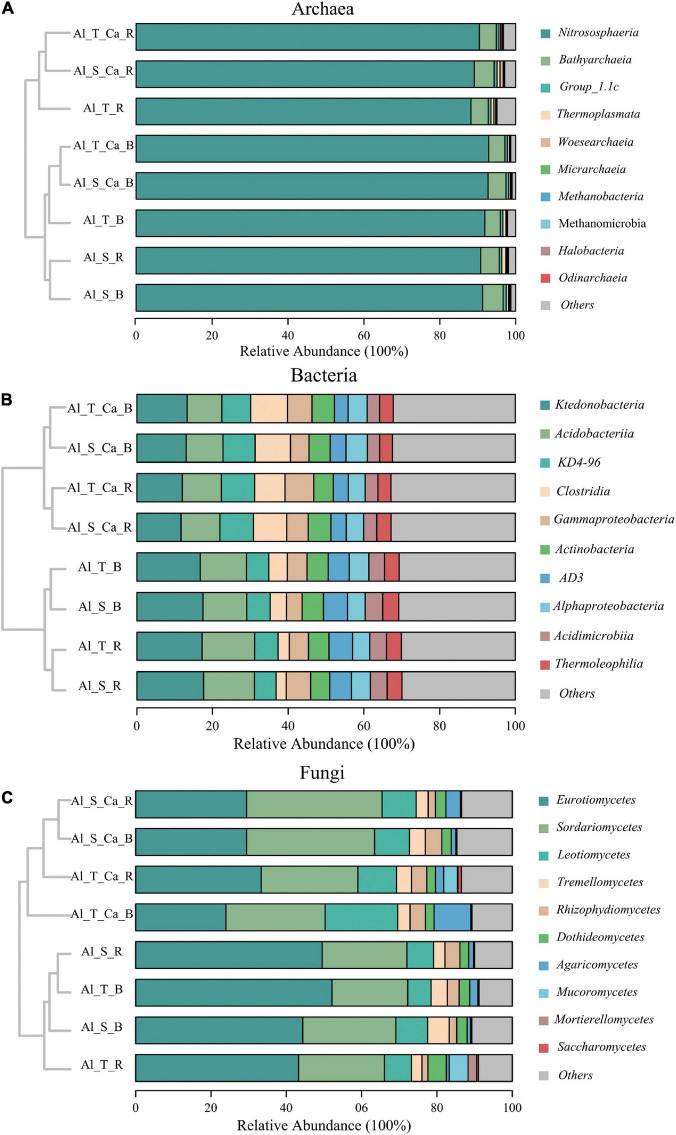
Hierarchical cluster analysis and relative abundance of the dominant archaea **(A)**, bacteria **(B)**, and fungi **(C)** in the bulk and rhizosphere soils under liming and non-liming conditions. Al_S_B, Al-sensitive rice bulk soil; Al_S_R, Al-sensitive rice rhizosphere soil; Al_T_B, Al-tolerant rice bulk soil; Al_T_R, Al-tolerant rice rhizosphere soil; Al_S_Ca_B, Al-sensitive rice and CaCO_3_ bulk soil; Al_S_Ca_R, Al-sensitive rice and CaCO_3_ rhizosphere soil; Al_T_Ca_B, Al-tolerant rice and CaCO_3_ bulk soil; Al_T_Ca_R, Al-tolerant rice and CaCO_3_ rhizosphere soil.

Across all soil samples, *Nitrososphaeria* (88–93%) was the most abundant archaeal class (Figure 3A). The main bacterial class, with a relative abundance exceeding 5%, included *Ktedonobacteria* (12–18%), *Acidobacteria* (9–14%), *KD4-96* (6–9%), *Clostridia* (3–10%), *Gammaproteobacteria* (4–7%), *Actinobacteria* (5–6%), and *AD3* (4–6%) (Figure 3B). In addition, 73–83% of the fungal sequences belonged to *Eurotiomycetes* (23–52%), *Sordariomycetes* (20–36%), *Leotiomycetes* (6–20%), and *Tremellomycetes* (3–6%) (Figure 3C). The hierarchical cluster analysis revealed that the archaeal, bacterial, and fungal community composition could be separated on the basis of liming (with or without lime) and soil compartments (bulk or rhizosphere soil), whereas rice genotypes (Al-sensitive or Al-tolerant) mainly affected the bacterial and fungal community composition (Figure 3).

**FIGURE 4:**
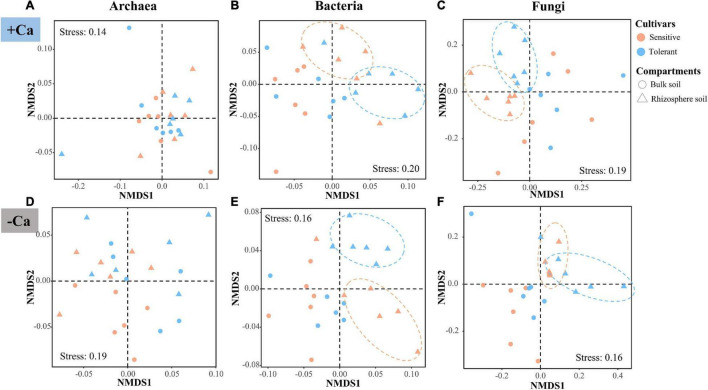
β-diversity of archaea **(A,D)**, bacteria **(B,E)**, and fungi **(C,F)** according to the Bray–Curtis dissimilarity matrix in soil samples under liming (+ Ca) and non-liming (−Ca) conditions.

The NMDS and PERMANOVA analyses of the ASVs revealed that the β-diversity of archaea, bacteria, and fungi differed substantially between the liming and non-liming conditions as well as between the bulk and rhizosphere soils, whereas there was no difference between the Al-tolerant and Al-sensitive genotypes across all soil samples (Supplementary Figure 2 and Supplementary Table 2). The strong effect of lime may have masked the effect of the rice genotypes on the β-diversity of microbial communities. Thus, we reanalyzed the β-diversity under liming and non-liming conditions. According to PERMANOVA, the β-diversity of the bacterial and fungal communities in the rhizosphere soil differed significantly between the two rice genotypes under liming or non-liming conditions (*P* < 0.05); a significant difference was not detected for the archaeal community (Figure 4 and Supplementary Table 3).

The Mantel test indicated that soil pH and exchangeable Ca were the main factors associated with changes in the community structure of archaea, bacteria, and fungi in bulk soil samples (Table 3). In addition, soil exchangeable Al was also strongly related to the community structure of archaea, bacteria, and fungi in rhizosphere soil samples. Hence, exchangeable Al appears to be another key factor associated with the variation in the microbial community structure in rhizosphere soils.

**TABLE 3:** Mantel test of the correlation between soil properties according to the Euclidean distance and the community structure of archaea, bacteria, and fungi according to the Bray–Curtis distance.

Environmental factors	Bulk soil						Rhizosphere soil					
	Archaea		Bacteria		Fungi		Archaea		Bacteria		Fungi	
	*r*	*P*	*r*	*P*	*r*	*P*	*r*	*P*	*r*	*P*	*r*	*P*
pH	0.275	**0.001**	0.833	**0.001**	0.234	**0.001**	0.193	**0.004**	0.811	**0.001**	0.343	**0.001**
AP	0.010	0.424	0.085	0.106	0.062	0.253	−0.066	0.669	0.043	0.263	−0.216	0.958
Mn	0.038	0.320	0.308	**0.002**	0.091	0.224	−0.024	0.567	−0.068	0.852	−0.020	0.541
Ca	0.236	**0.001**	0.685	**0.001**	0.102	**0.044**	0.300	**0.001**	0.864	**0.001**	0.447	**0.001**
Mg	−0.027	0.624	−0.064	0.903	−0.093	0.899	−0.055	0.665	−0.055	0.776	−0.076	0.708
Al	0.117	0.072	0.624	**0.001**	0.070	0.192	0.241	**0.001**	0.822	**0.001**	0.341	**0.001**
NH_4_^+^-N	−0.027	0.579	−0.031	0.629	−0.035	0.619	0.077	0.217	0.116	0.061	−0.115	0.868
NO_3_^–^-N	0.029	0.346	0.154	0.032	0.249	0.318	−0.081	0.750	−0.052	0.773	−0.101	0.793

*Significant correlations (P < 0.05) are indicated (i.e., bold values).*

*AP, available P.*

### Genotype-Specific Microbial Taxa in Rhizosphere Soils

**FIGURE 5:**
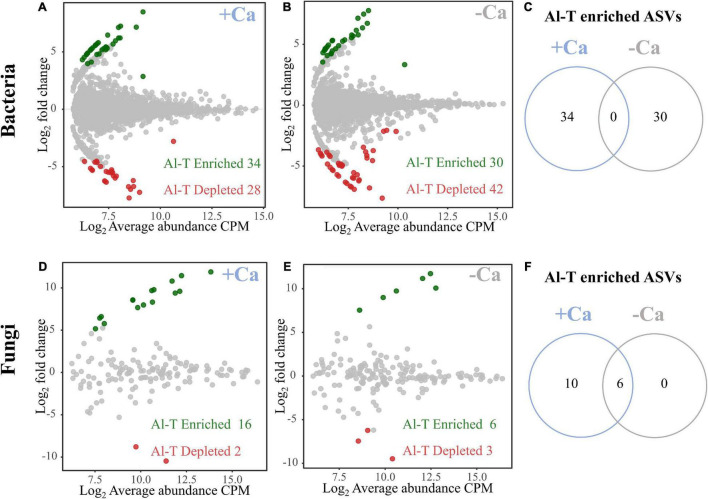
Special sets of bacteria **(A–C)** and fungi **(D–F)** enriched and depleted in the rhizosphere of the Al-tolerant rice genotype under liming (+ Ca) and non-liming (−Ca) conditions. The MA plots present the average abundance (in log count per million, CPM) and the log-fold change of all ASVs plotted on the *x*-axis and *y*-axis, respectively. Differentially enriched and depleted ASVs were determined on the basis of an edgeR analysis (FDR < 0.05, | log_2_ fold change| ≥ 2). Green and red, respectively, indicate enriched and depleted bacterial and fungal OTUs. The number of enriched bacterial **(C)** and fungal **(F)** ASVs in the Al-tolerant rice genotype is compared between the liming (+ Ca) and non-liming (−Ca) conditions.

To analyze the genotype-specific microbial taxa in rhizosphere soils under liming or non-liming conditions, we searched for bacterial and fungal ASVs that differed significantly in terms of their relative abundance between the Al-tolerant and Al-sensitive genotypes. Under the liming condition, we detected 62 genotype-specific bacterial ASVs (34 enriched and 28 depleted in the Al-tolerant genotype) and 18 genotype-specific fungal ASVs (16 enriched and two depleted in the Al-tolerant genotype) (Figures 5A,D). Under the non-liming condition, we detected 72 genotype-specific bacterial ASVs (30 enriched and 42 depleted in the Al-tolerant genotype) and nine genotype-specific fungal ASVs (six enriched and three depleted in the Al-tolerant genotype) (Figures 5B,E). The enriched bacterial ASVs were mainly affiliated with *Proteobacteria*, *Chloroflexi*, *Actinobacteria*, and *Firmicutes*, whereas the enriched fungal ASVs were mainly affiliated with *Mucoromycota* and *Ascomycota* (Supplementary Table 4). None of the enriched bacterial ASVs in the Al-tolerant genotype were detected under both non-liming and liming conditions (Figure 5C). In contrast, six enriched fungal ASVs were common to both non-liming and liming conditions (Figure 5F).

### Predicted Factors Contributing to the Rice Biomass

**FIGURE 6:**
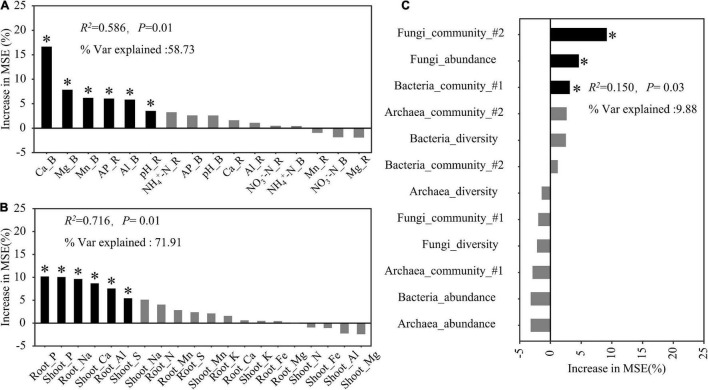
Predicted factors, soil properties **(A)**, plant mineral elements **(B)**, and rhizosphere microbial properties **(C)**, contributing to the total rice biomass following a random forest regression analysis. *R*^2^, coefficient of determination; % Var explained, the proportion of variance explained. The microbial diversity and community used in this model were determined according to the Shannon index and the first two axes (#1 and #2) of the non-metric multidimensional scaling analysis, respectively. * Indicates factors with significant effects (*P* < 0.05).

The random forest regression analysis revealed that soil properties and plant mineral elements explained 58.73 and 71.91% of the total variation in the rice biomass, respectively (Figures 6A,B). Among the soil factors, soil exchangeable Ca explained the largest proportion of this variation, but other soil factors, including Mg, Mn, and Al in the bulk soil as well as pH and available P in the rhizosphere soil, also significantly contributed to the variation in the rice biomass (Figure 6A). The shoot and root P contents were the most important factors explaining the variation in the rice biomass, followed by the root Na, shoot Ca, and root Al contents (Figure 6B). Microbial properties explained 9.88% of the total variation in the rice biomass, of which fungal community structure accounted for the largest proportion of this variation, followed by fungal abundance and bacterial community structure (Figure 6C).

### Comparison of Microbial Co-occurrence Networks

**FIGURE 7:**
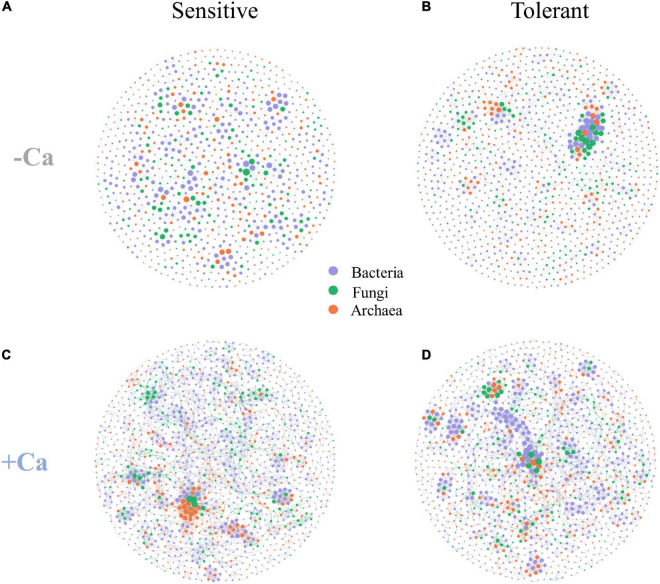
Co-occurrence networks of archaeal, bacterial, and fungal communities for Al-sensitive **(A,C)** and Al-tolerant **(B,D)** rice genotypes in acid sulfate soils under liming and non-liming conditions. The size of each node is proportional to the number of connections. The archaeal, bacterial, and fungal ASVs are indicated in orange, purple, and green, respectively.

Four microbial co-occurrence networks were constructed for the Al-sensitive and Al-tolerant rice genotypes under non-liming and liming conditions (Figure 7). Compared with the Al-sensitive rice genotype, the Al-tolerant rice genotype had a more complex microbial network with higher values for the nodes, edges, graph density, average node degree, average clustering coefficient, and keystone ASVs under the non-liming condition (Figures 7A,B and Table 4). Compared with the non-liming condition, liming increased the complexity of the microbial networks of both rice genotypes (Figure 7 and Table 4). Under the liming condition, the microbial networks of the Al-sensitive and Al-tolerant rice genotypes tended to be comparable (Figures 7C,D and Table 4).

**TABLE 4:** Topological properties of co-occurring networks and the number of keystone ASVs.

Network metrics	Al-S	Al-T	Al-S + Ca	Al-T + Ca
Nodes	937	1,060	1,267	1,196
Edges	1,564	2,516	3,228	2,819
Network diameter	20	29	37	30
Graph density	0.0036	0.0045	0.0040	0.0039
Average node degree (avgK)	3.338	4.747	5.096	4.714
Average clustering coefficient (avgCC)	0.687	0.706	0.705	0.708
Average path length (APL)	5.844	7.036	14.345	10.534
Modularity (M)	0.962	0.921	0.94	0.95
Keystone ASVs	13	86	76	74

*Al-S, Al-sensitive rice; Al-T, Al-tolerant rice; Al-S + Ca, Al-sensitive rice and CaCO_3_; Al-T + Ca, Al-tolerant rice and CaCO_3_.*

## Discussion

### Liming Was the Main Factor Altering the Microbial Diversity and Community Structure

Soil pH is a key factor affecting the soil microbial community. In this study, liming, which increased the soil pH and decreased Al toxicity, affected the β-diversity of archaea, bacteria, and fungi. This finding can be attributed to the effects of liming on soil properties (e.g., pH as well as exchangeable Ca and Al contents). The microbial community composition is determined by soil chemical properties (Bulgarelli et al., 2013). The Mantel test results here indicated that soil pH and exchangeable Ca and Al contents were closely associated with the shifts in the community structure of archaea, bacteria, and fungi. Furthermore, liming affected the α-diversity of only bacteria and fungi, implying that the α-diversity of bacteria and fungi may be more sensitive to soil pH than that of archaea. The low sensitivity of archaea to liming in this study may be explained by the fact the identified archaea were mainly affiliated with *Nitrososphaeria* (89–93%), which has a chemolithoautotrophic lifestyle and is highly adaptable to acidic soils (Wang et al., 2014; Lehtovirta-Morley et al., 2016). Although liming had some observable effects on microbial α-diversity and β-diversity, it did not alter the abundance of archaea, bacteria, and fungi, probably because of the short culture period in this study.

### The Rice Genotypes Shifted the Community Structure of Bacteria and Fungi in the Absence of Liming

The rhizosphere had significant effects on microbial β-diversity, but it did not affect microbial α-diversity and abundance in this study. Changes in the microbial community composition may not necessarily result in changes in α-diversity and abundance because the shifts in some microbial taxa may be compensated by changes in other microbial taxa (Hartmann and Widmer, 2006). In this study, there were no significant differences in the overall abundance, α-diversity, and β-diversity of archaea, bacteria, and fungi between the Al-tolerant and Al-sensitive rice genotypes across all soil samples. However, when the liming effect was eliminated, the rice genotypes significantly affected the bacterial and fungal community structure in the rhizosphere under non-liming and liming conditions. In addition, the bacterial and fungal communities were clustered in two groups according to rice Al tolerance. This is consistent with the findings of previous studies on soybean (Yang et al., 2012b; Lian et al., 2019; Shi et al., 2020a,b). We observed that the archaeal community structure was unaffected by rice genotypes, likely because of the low sensitivity of archaea to the study environmental conditions (as described above). We also revealed that the community structure of fungi and bacteria, but not of archaea, was mainly responsible for the variation in the rice biomass. These results reflect the importance of bacteria and fungi for rice growth in acid sulfate soils.

### The Aluminum-Tolerant Rice Genotype Recruited Specific Bacterial and Fungal Taxa in the Rhizosphere

Soil microorganisms can improve plant productivity and nutrient absorption both directly and indirectly by altering soil nutrient availability or promoting plant growth (Pii et al., 2015). Recent research demonstrated that soil microbial keystone taxa can drive crop productivity (Zhong et al., 2020; Fan et al., 2021; Wang et al., 2021, 2022). In this study, we analyzed the genotype-specific microbial taxa in rhizosphere soils on the basis of differential ASV abundance. Several ASVs from bacteria (mainly *Proteobacteria*, *Firmicutes*, and *Actinobacteria*) and fungi (mainly *Mucoromycota* and *Ascomycota*) were specifically enriched in the rhizosphere of Al-tolerant rice. These microbial taxa were also detected in the rhizosphere of an Al-tolerant soybean accession in earlier studies (Yang et al., 2012b; Lian et al., 2019; Shi et al., 2020b). Notably, in this study, some plant growth-promoting bacteria (i.e., *Bacillus* and *Pseudomonas*) were enriched in the rhizosphere of only the Al-tolerant rice genotype under the non-liming condition (Supplementary Table 4). Some *Bacillus* species are highly tolerant to acidic conditions and can solubilize mineral P (Çakmakçı et al., 2010). Some *Pseudomonas* species are able to promote plant growth by enhancing the secretion of organic acids from their roots and increasing soil P bioavailability (Phillips et al., 2004; Sharma et al., 2011; Otieno et al., 2015). Moreover, the Al-tolerant rice genotype in this study consistently enriched some fungi (i.e., *Aspergillus* and *Rhizopus*) in the rhizosphere under both liming and non-liming conditions (Supplementary Table 4). *Aspergillus* and *Rhizopus* are phosphate-solubilizing microorganisms that can enhance plant P uptake and promote plant growth (Alori et al., 2017). Soil P deficiency and Al toxicity are the two main factors limiting crop yield in acidic soils (Zheng, 2010; Chen et al., 2012; Chen and Liao, 2016). In this study, the Al-tolerant rice genotype was better able to take up P from soils than the Al-sensitive rice genotype. In addition, the P contents of soils and rice plants were the primary factors associated with the variation in the rice biomass. In a recent study, we observed that *Nguyenibacter* sp. L1 isolated from the rhizosphere of the Al-tolerant plant *Lespedeza bicolor* solubilizes fixed P and alleviates the toxic effects of Al on rice by secreting organic acids (Li et al., 2021). The results of these analyses collectively suggest that the specific bacterial and fungal taxa enriched in the rhizosphere of Al-tolerant rice genotypes may potentially influence the secretion of organic acids by plants and P solubilization as well as promote plant growth, thereby modulating rice Al tolerance and P uptake efficiency. These changes ultimately result in increased rice plant growth in acid sulfate soil. The specific microbial taxa recruited by Al-tolerant rice genotypes may be useful for the management of acid sulfate soil and as indicators of rice genotypes applicable for breeding Al-tolerant cultivars. A similar strategy may be applied to improve the tolerance of important crops to various environmental stresses.

### The Aluminum-Tolerant Rice Genotype Had a More Complex Soil Microbial Co-occurrence Network Than the Aluminum-Sensitive Rice Genotype

The microbiome structure is closely associated with microbial functions. Microbial co-occurrence network analyses have provided researchers with important insights into microbial structures and their response to environmental stress (Edwards et al., 2015; Ramirez et al., 2018). In our study, the rice genotypes significantly affected microbial co-occurrence networks. The Al-tolerant rice genotype had a more complex microbial co-occurrence network and more keystone ASVs than the Al-sensitive genotype under the non-liming condition, which may reflect the relative stability of the microbial network of the Al-tolerant genotype under Al stress conditions. Because different microbial taxa can complement each other (Banerjee et al., 2019), certain microbial taxa recruited by the Al-tolerant genotype may mitigate Al toxicity by promoting the cooperation and competition among the microbes in the network. Interestingly, liming increased the network complexity for both genotypes but also decreased the differences in the network between the two genotypes. This can be attributed to the decreased exchangeable Al content in the soil, which improved the environmental conditions for the growth of rice plants and microbes and subsequently increased the interactions among soil microbes.

## Conclusion

The microbial community composition in the rhizosphere is associated with the Al tolerance of rice plants in acid sulfate soil. Rice Al tolerance was observed to be related to changes in the community structure of bacteria and fungi in the rhizosphere. The Al-tolerant rice genotype recruited several specific bacterial and fungal taxa (i.e., *Bacillus*, *Pseudomonas*, *Aspergillus*, and *Rhizopus*) that are P-solubilizing and plant growth-promoting microbes. Moreover, the Al-tolerant rice genotype had a more complex microbial co-occurrence network in the rhizosphere than the Al-sensitive genotype. Aluminum toxicity and P deficiency are the two primary factors inhibiting plant growth in acidic soil. The altered microbial community structure, recruitment of specific taxa, and the complex microbial network of Al-tolerant rice genotypes might enhance rice plant Al tolerance and P uptake, ultimately contributing to the improved growth of Al-tolerant rice in acid sulfate soil. Therefore, our study revealed the ecological link between rice Al tolerance and the soil microbiota, which has important implications for increasing agricultural productivity and enhancing ecosystem functioning in regions with acid sulfate soil. Due to the limitation of soil volume under the pot experiment, it is important to further explore the link between rice Al tolerance and soil microbiota under the field condition.

## Data Availability Statement

The datasets presented in this study can be found in online repositories. The names of the repository/repositories and accession number(s) can be found in the article/Supplementary Material.

## Author Contributions

XZ contributed to the study conception and design. XX, JW, JL, XL, and XD performed the experiments. XX analyzed the data, prepared the figures and tables, and wrote the manuscript draft. XZ, RS, and JW revised the manuscript. All authors read and approved the final manuscript.

## Conflict of Interest

The authors declare that the research was conducted in the absence of any commercial or financial relationships that could be construed as a potential conflict of interest.

## Publisher’s Note

All claims expressed in this article are solely those of the authors and do not necessarily represent those of their affiliated organizations, or those of the publisher, the editors and the reviewers. Any product that may be evaluated in this article, or claim that may be made by its manufacturer, is not guaranteed or endorsed by the publisher.

## Footnotes

1
https://www.arb-silva.de/

2
https://unite.ut.ee/

3
http://rdp.cme.msu.edu/
