# Supplementary material for: Distinct Patterns of Rhizosphere Microbiota Associated With Rice Genotypes Differing in Aluminum Tolerance in an Acid Sulfate Soil
Source: Front Microbiol. 2022 Jun 17;13:933722. doi: 10.3389/fmicb.2022.933722 (PMC9247542; doi:10.3389/fmicb.2022.933722)
Supplement: Supplementary file 1 [file Data_Sheet_1.docx]

**Supplementary Figures**


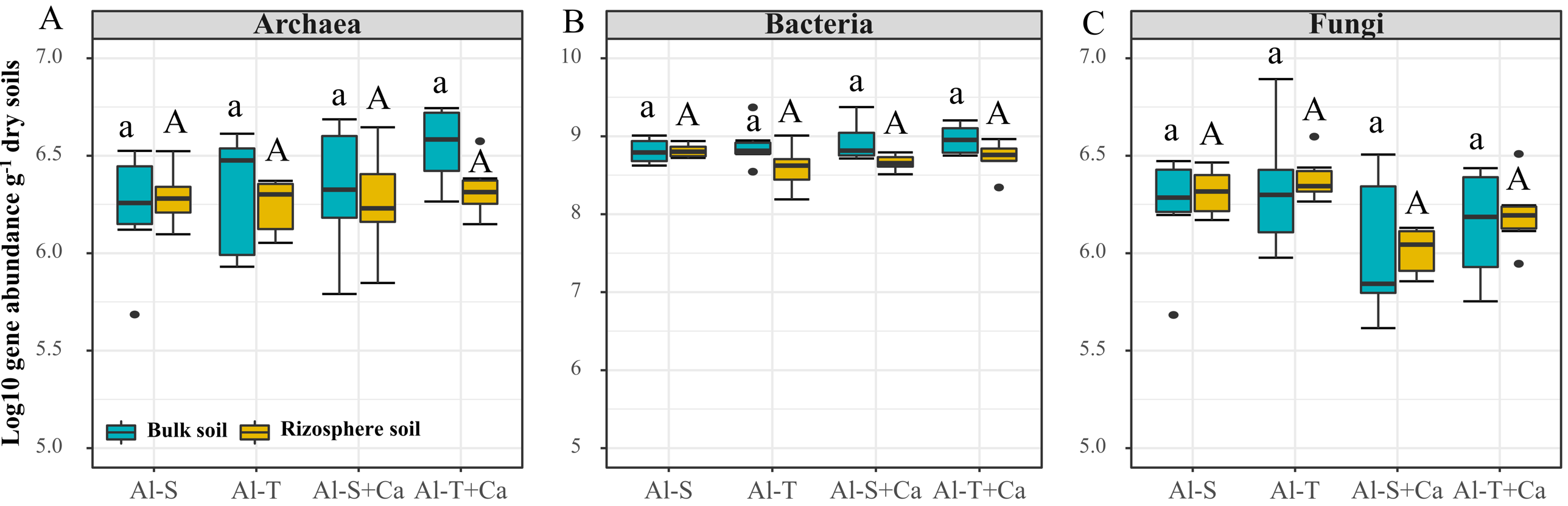


**Fig. S1** Abundance of archaea (**a**), bacteria (**b**), and fungi (**c**) under different treatment conditions. Al-S, Al-sensitive rice; Al-T, Al-tolerant rice; Al-S+Ca, Al-sensitive rice and CaCO_3_; Al-T+Ca, Al-tolerant rice and CaCO_3_


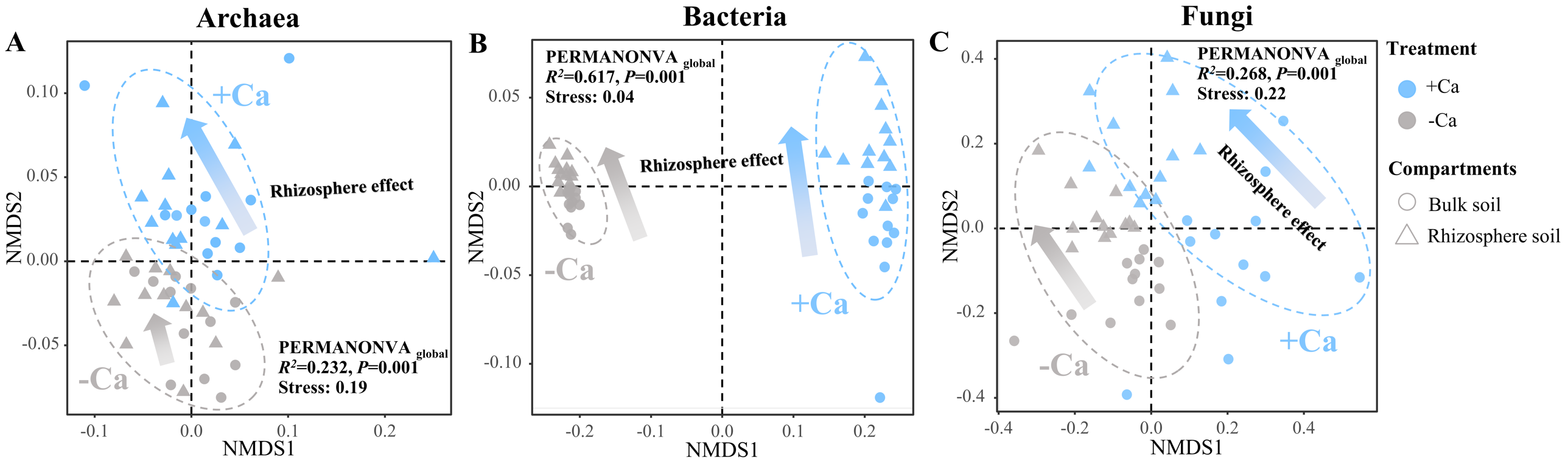


**Fig. S2** Variation in the community structure of archaea (**a**), bacteria (**b**), and fungi (**c**) across all soil samples according to a non-metric multidimensional scaling analysis involving the Bray–Curtis dissimilarity matrix

**Supplementary Tables**

| Items | Primer | Thermal cycling condition | Reference |
| --- | --- | --- | --- |
| Archaea | 524F: TGYCAGCCGCCGCGGTAA | 94 °C, 5 min; 35 × (94 °C for 1 min, 56 °C for 1 min, 72 °C for 1 min） | Pires et al. (2012) |
|  | 958R: YCCGGCGTTGAVTCCAATT |  |  |
| Bacteria | 515F: GTGYCAGCMGCCGCGGTAA | 95 °C, 5 min; 35 × (95 °C for 30 s, 58 °C for 30 s, 72 °C for 1 min） | Walters et al. (2016) |
|  | 926R: CCGYCAATTYMTTTRAGTTT |  |  |
| Fungi | gITS7: GTGARTCATCGARTCTTTG | 94 °C, 5 min; 30 × (98 °C for 10 s, 55 °C for 30 s, 72 °C for 30 s） | Ihrmark et al. (2012) |
|  | ITS4: TCCTCCGCTTATTGATATGC |  |  |

**Table S1** Primers and thermal cycling conditions used for high-throughput sequencing

**Table S2** Global effect of soil conditions (with or without liming), soil compartments (bulk and rhizosphere), and rice genotypes (Al-sensitive and Al-tolerant) on the β-diversity of archaea, bacteria, and fungi as determined by PERMANOVA

| Item |  | Archaea | |  | Bacteria | |  | Fungi | |
| --- | --- | --- | --- | --- | --- | --- | --- | --- | --- |
|  |  | *R*^2^ | *P* |  | *R*^2^ | *P* |  | *R*^2^ | *P* |
| Liming VS Non-liming |  | **0.099** | **0.001** |  | **0.503** | **0.001** |  | **0.063** | **0.001** |
| Bulk soil VS Rhizosphere soil |  | **0.040** | **0.014** |  | **0.037** | **0.012** |  | **0.080** | **0.001** |
| Al-sensitive VS Al-tolerant |  | 0.026 | 0.149 |  | 0.017 | 0.137 |  | 0.024 | 0.111 |

Significant differences (*P* < 0.05) are indicated (i.e., bold values).

**Table S3** Effect of rice genotypes on the bacterial and fungal community in the rhizosphere soil under liming or non-liming conditions as determined by PERMANOVA

| Treatments | Item |  | Archaea | |  | Bacteria | |  | Fungi | |
| --- | --- | --- | --- | --- | --- | --- | --- | --- | --- | --- |
|  |  |  | *R*^2^ | *P* |  | *R*^2^ | *P* |  | *R*^2^ | *P* |
| +Ca | Al-sensitive VS Al-tolerant |  | 0.076 | 0.674 |  | **0.140** | **0.026** |  | **0.143** | **0.010** |
| -Ca | Al-sensitive VS Al-tolerant |  | 0.091 | 0.389 |  | **0.143** | **0.024** |  | **0.124** | **0.035** |

Significant differences (*P* < 0.05) are indicated (i.e., bold values).

**References for supplemental materials**

New primers to amplify the fungal ITS2 region–evaluation by 454-sequencing of artificial and natural communities. *FEMS Microbiol. Ecol.* 82, 666–677. doi:10.1111/j.1574-6941.2012.01437.x

Pires, A. C., Cleary, D. F., Almeida, A., Cunha, Â., Dealtry, S., Mendonça-Hagler, L. C., et al. (2012). Denaturing gradient gel electrophoresis and barcoded pyrosequencing reveal unprecedented archaeal diversity in mangrove sediment and rhizosphere samples. *Appl. Environ. Microbiol.* 78, 5520–5528. doi:10.1128/AEM.00386-12

Walters, W., Hyde, E. R., Berg-Lyons, D., Ackermann, G., Humphrey, G., Parada, A., et al. (2016). Improved bacterial 16S rRNA gene (V4 and V4-5) and fungal internal transcribed spacer marker gene primers for microbial community surveys. *Msystems* 1, e00009–15. doi:10.1128/mSystems.00009-15
